# Dynapenic abdominal obesity and susceptibility to fall: a prospective analysis of the Osteoarthritis Initiative

**DOI:** 10.3389/fnut.2023.1153399

**Published:** 2023-05-05

**Authors:** Nicola Veronese, Ai Koyanagi, Pinar Soysal, Francesco Bolzetta, Ligia J. Dominguez, Mario Barbagallo, Shaun Sabico, Nasser M. Al-Daghri, Lee Smith

**Affiliations:** ^1^Department of Health Promotion, Mother and Child Care, Internal Medicine and Medical Specialties, University of Palermo, Palermo, Italy; ^2^Research and Development Unit, Parc Sanitari Sant Joan de Déu, CIBERSAM, ISCIII, Barcelona, Spain; ^3^ICREA, Barcelona, Spain; ^4^Department of Geriatric Medicine, Faculty of Medicine, Bezmialem Vakif University, Istanbul, Turkey; ^5^Medical Department, Geriatric Unit, Azienda ULSS (Unità Locale Socio Sanitaria) 3 "Serenissima", Venice, Italy; ^6^Faculty of Medicine and Surgery, Kore University of Enna, Enna, Italy; ^7^Chair for Biomarkers of Chronic Diseases, Biochemistry Department, College of Science, King Saud University, Riyadh, Saudi Arabia; ^8^Centre for Health, Performance, and Wellbeing, Anglia Ruskin University, Cambridge, United Kingdom

**Keywords:** abdominal obesity, dynapenia, dynapenic abdominal obesity, falls, older adults, Osteoarthritis Initiative

## Abstract

**Background:**

The prediction of the risk of falling remains a challenge in geriatric medicine and the identification of new potential reversible risk factors is a public health priority. In this study, we aim to investigate the association between DAO (dynapenic abdominal obesity) and incident falls in a large sample of people with knee OA (osteoarthritis) or at high risk for this condition, over 8 years of follow-up.

**Methods:**

DAO was defined using a waist circumference more than 102 cm in men and 88 cm in women and a concomitant presence of dynapenia, defined as a time over 15 s in the five times chair stands time. Falls, during follow-up, were recorded using self-reported information in the previous year. A logistic binary regression analysis was run, adjusted for potential confounders at the baseline, reporting the data as odds ratios (ORs) with their 95% confidence intervals (CIs).

**Results:**

Overall, 3,844 subjects were included, majority of whom had abdominal obesity. Across the 8 years of follow-up, 2,695 participants fell vs. 1,149 not reporting any fall. Taking those without DAO as reference, the presence of only dynapenia was not associated with risk of falls (OR = 1.18;95%CI: 0.73–1.91; *p* = 0.50), whilst the presence of abdominal obesity (OR = 1.30; 95%CI: 1.09–1.56; *p* = 0.004) and DAO (OR = 1.31; 95%CI:1.01–1.73; *p* = 0.04) were significantly associated with a higher risk of incident falls.

**Conclusion:**

DAO significantly increased risk of falls as well as the presence of abdominal obesity.

## 1. Introduction

Falls are a major public health concern in older people as they are associated with worse quality of life, increased physical comorbidity, healthcare use and early mortality (1). It has been estimated that approximately 1 in 3 people aged 65 years or older fall every year in the United States (US) (2). Moreover, in the US, falls are a frequent cause of disability, institutionalization, and mortality, and are among the primary causes of traumatic injury among the older adult population (1, 2). Several conditions are known as potential risk factors for falls, including reduction in muscle strength (3), gait imbalance (4), visual and hearing deficits (5), osteoarthritis (6), dementia (7), and depression (8). The early identification of older adults who are at risk of falling is important in order to develop tailored interventions to prevent falls (9). However, the prediction of such a risk of falling remains a challenge in geriatric medicine (10).

While many risk factors for falls have been identified to date (11), one potentially important but understudied risk factor is dynapenic abdominal obesity (DAO), usually defined as the impairment in muscle strength and high waist circumference (12). Those with DAO are more likely to experience decreased postural stability caused by abdominal fat accumulation and this in turn increases fall risk (12). Moreover, the impaired muscular system in DAO may lead to difficulties responding to postural correction with sufficient strength and speed, subsequently resulting in falls (13). In one large-scale longitudinal study in UK involving 4,987 individuals ≥60 years old and followed up for of 14-years, it was observed that DAO was significantly associated with higher risk for falls, recurrent falls, or injurious falls by as much as significant 1.2–1.3 times as compared to those without DAO (14). A similar but smaller longitudinal study in Brazil (*n* = 201 females) with 18-months of follow-up also found that DAO was significantly associated with risk of falls (3.6 times) than those without DAO (15). Other studies utilizing a cross-sectional design have found similar findings (14, 16). To the best of the authors’ knowledge, no other studies exist on this topic. More longitudinal research is needed to confirm or refute findings of the two previous longitudinal studies, of which one has limited generalizability as it only included a small sample of women.

Importantly, those with knee osteoarthritis (OA) or at high risk of this condition may be at a greater risk of DAO, independent from already known morbid outcomes such as falls and cardiovascular disease (17). For example, central obesity has been implicated in the development and progression of knee OA (18), while those with OA have been reported to have a weak handgrip strength (19). Moreover, those with knee OA are at a higher risk of falling owing to impaired balance, muscle weakness, presence of comorbidities, and increasing number of symptomatic joints (20). It would thus be prudent to examine the relationship between DAO and incidence falls in this high-risk population.

Given this background, the aim of the present study was to investigate the association between DAO and incident falls in a large sample of people with knee OA or at high risk for this condition, over 8 years of follow-up.

## 2. Materials and methods

### 2.1. Data source and subjects

The OAI cohort study is a multi-center, longitudinal, observational study focusing primarily on knee OA (21). Participants, with an age between 40 and 80 years, were recruited across four clinical sites in the United States of America (Baltimore, MD; Pittsburgh, PA; Pawtucket, RI; and Columbus, OH) between February 2004 and May 2006.

The OAI created a public archive of data, biological samples, and joint images collected over time from a very well clinically characterized population of individuals comprised of two subgroups,: (1) had knee osteoarthritis (OA) with knee pain for a 30-day period in the past 12 months or (2) were at high risk of developing knee OA (e.g., overweight/obese (body mass index, BMI ≥25 kg/m^2^), family history of knee OA). The baseline assessments consist of an initial eligibility assessment by telephone, a screening clinic visit and an enrollment clinic visit. Several follow-up visits were repeated.

The data of this longitudinal cohort study were collected at baseline and during subsequent evaluations, with a follow-up of 8 years. All participants provided written informed consent. The OAI study was given full ethics approval by the institutional review board of the OAI Coordinating Center, at the University of California in San Francisco, CA, USA.

### 2.2. Exposure: dynapenic abdominal obesity

As measure of muscle strength, we used the five times chair stands time. According to the most recent guidelines on sarcopenia, dynapenia was defined using a value, in both genders, more than 15 s (22). We considered a subject dynapenic if he/she score more than 15 s in either of the two attempts. For waist circumference, the waist was defined as the point midway between the iliac crest and the costal margin (lower rib). The tape was kept horizontal and the measurement to the nearest mm was taken. Abdominal obesity was defined as a waist circumference of >88 cm in women and > 102 in men (23). Accordingly, participants were divided into four groups, i.e., no dynapenia and no abdominal obesity [reference], dynapenia alone, abdominal obesity alone, and DAO.

### 2.3. Outcome: incidence of falls

A fall is traditionally defined as “an event which resulted in a person coming to rest inadvertently on the ground or floor or other lower level” (24).

The assessment of the outcome was carried out at baseline and during the follow-up visits at 12, 24, 36, 48, 72, and 96 months. At the end of each wave, including baseline evaluation, participants reported the number of falls experienced in the preceding year by answering the question: “Did you fall during the past year?” The number of falls was also recorded. No information was available regarding the date of the fall.

### 2.4. Control variables

In the association between DAO and incident falls, we considered several factors at the baseline evaluation, including: demographic characteristics (age, sex, ethnicity); education level (categorized as college vs. lower grades); smoking status (categorized as actual vs. never/previous); yearly income (more or less than 50,000$); number of alcoholic drinks consumed during a typical week in the last month; physical activity was measured through the Physical Activity Scale for the Elderly scale (PASE) (25); the presence and severity of comorbidities assessed by the Charlson Comorbidity Index score (26); number of medications used; presence, at the baseline, of previous falls. The OAI database includes bilateral posteroanterior fixed-flexion knee radiographs from patients. Semiquantitative Kellgren and Lawrence (KL) Grades were used for grading the severity of knee OA (27). The images were centrally graded by two expert readers who were blinded to each other’s readings and to clinical data from the patient (28).

### 2.5. Statistical analyses

Data on continuous variables were normally distributed according to the Kolmogorov–Smirnov test. Data were presented as means and standard deviation values (SD) for quantitative measures (if normally distributed) or as medians and interquartile ranges (if not normally distributed), and percentages for all categorical variables by incidence of falls during follow-up period (yes vs. no). *p*-values were calculated with the Fisher’s Exact test for frequencies, Mann–Whitney test for medians and independent t-Test for means.

Logistic binary regression analysis was run, considering as outcome the incidence of falls during follow-up and as main exposures the presence, at the baseline, of dynapenia and/or abdominal obesity status. Similarly, we reported the associations found for the other control variables. The strength of the association between factors at the baseline and the outcomes of interest were reported as odds ratios (ORs) with their 95% confidence intervals (CIs). To the test the robustness of our results, we did run several sensitivity analyses (e.g., DAO by sex, age, race, presence at the baseline of knee OA and its severity and other covariates cited in paragraph 2.4), but all these interaction reported a *p*-value >0.05.

A *p* < 0.05 was deemed statistically significant. Analyses were performed using SPSS 26.0.

## 3. Results

Among the 4,796 participants initially included, 943 did not have sufficient information regarding chair stands time or waist circumference, making it not possible to allocated them to any of the four categories of DAO and nine did not report any information regarding incident falls. Therefore, 3,844 subjects were included. Overall, at the baseline, participants without abdominal obesity and dynapenia included 27.5% of the population, whilst abdominal obesity affected 58.8% of the participants and dynapenia 2.5%. Finally, the co-presence of DAO affected 11.2% of the population examined at the baseline. As expected, the prevalence of radiological knee OA was significantly higher in DAO (79.1%) compared to only obesity (64.6%) and only dynapenia (62.1%) than in people without these two conditions (51.2%; *p* < 0.0001 for the comparison).

Table 1 shows the baseline characteristics by incident falls during the 8 years of follow-up. Compared to participants not reporting any fall during follow-up (*n* = 1,149) incident fallers (*n* = 2,695) were more likely to be female (*p* < 0.0001), but no differences in terms of age was observed (*p* = 0.21). Incident fallers reported a significantly higher number of medications (*p* < 0.0001), but a similar rate of comorbidities (*p* = 0.16). Moreover, they were more depressed and reported a higher presence of previous falls at the baseline (*p* < 0.0001 for both comparisons).

**Table 1:** Baseline characteristics, by incident falls during follow-up.

Characteristic	Categories	No incident falls (*n* = 1,149)	Incident falls (*n* = 2,695)	*p*-value^a^
Sex (%)	Females	48.7	60.5	<0.0001
Age (years)	Mean (SD)	60.9 (9.1)	61.3 (9.1)	0.21
Whites (%)	Yes	76.7	83.2	<0.0001
Education	College	29.3	32.3	0.07
Active smoker (%)	Yes	45.0	46.8	0.33
Yearly income (%)	>50,000$	34.6	35.2	0.73
Number of alcoholic drinks in a typical week	Mean (SD)	1.68 (1.46)	1.78 (1.48)	0.06
Physical activity scale for the Elderly (PASE)	Mean (SD)	162 (82)	164 (82)	0.68
Charlson comorbidity score	Mean (SD)	0.34 (0.80)	0.38 (0.82)	0.16
Number of medications	Mean (SD)	2.55 (2.50)	3.02 (2.69)	<0.0001
Center for Epidemiologic Studies Depression Scale (CES-D)	Mean (SD)	5.6 (5.9)	6.6 (6.9)	<0.0001
Radiological knee OA (%)	Yes	61.7	62.8	0.52
Grade 1 KL knee OA	Yes	28.6	29.6	0.33
Grade 2 KL knee OA	Yes	45.9	43.5	
Grade 3 KL knee OA	Yes	19.3	21.8	
Grade 4 KL knee OA	Yes	6.2	5.0	
Previous falls (%)	Yes	15.9	39.9	<0.0001
Chair stands time, s	Mean (SD)	11.4 (3.4)	11.5 (3.8)	0.15
Dynapenia (%)	Yes	12.5	14.1	0.19
Waist circumference, cm	Mean (SD)	101 (12)	102 (13)	<0.0001
Abdominal obesity (%)	Yes	63.8	72.6	<0.0001

Data are reported as percentages or as means with standard deviations. All data were obtained at the baseline evaluation.

a *p*-value was estimated by Chi-squared test and independent Student T-test for categorical and continuous variables, respectively.

**Figure 1:**
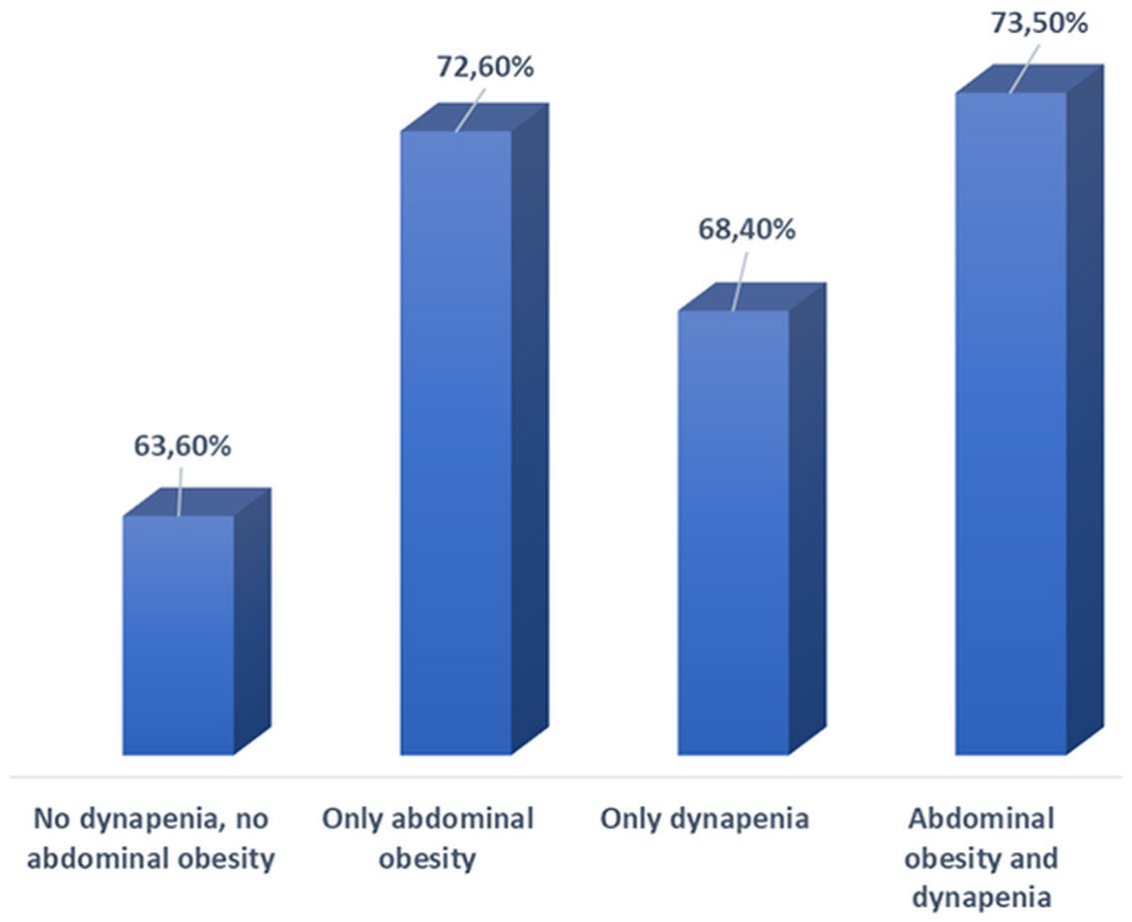
Incidence of falls at follow-up by DAO status at baseline. Incident falls (outcome) were those which were assessed during the follow-up period and referred to those that occurred since the baseline evaluation.

Among the items constituting DAO, incident fallers reported a significantly higher prevalence of abdominal obesity (72.6 vs. 63.8%, *p* < 0.0001), but not of dynapenia (*p* = 0.19; Table 1). This leads to the data shown in Figure 1 in which participants without abdominal obesity and dynapenia reported the lowest incidence of falls (63.6%), followed by only dynapenia (68.4%), abdominal obesity (72.6%), and DAO (73.5%).

Table 2 shows the logistic regression analysis adjusted for the potential confounders at the baseline. Taking those without DAO as reference, the presence of only dynapenia was not associated with any increased risk of falls (OR = 1.18;95%CI: 0.73–1.91; *p* = 0.50), whilst the presence of abdominal obesity (OR = 1.30; 95%CI: 1.09–1.56; *p* = 0.004) and dynapenia and abdominal obesity (OR = 1.31; 95%CI: 1.01–1.73; *p* = 0.04) were associated with a higher risk of incident falls. Among the other factors considered, being females, white, and highly educated were associated with a higher risk of falling during the follow-up. Moreover, higher values of alcohol drinking in a typical week, CES-D, PASE, number of medications as well as falls reported before the baseline had a significant higher risk of falling during the 8 years follow-up.

**Table 2:** Prospective association between dynapenia, abdominal obesity, or both at baseline and falls at follow-up estimated by multivariable logistic regression.

Characteristic		OR	95%CI	*p*-value
DAO status	No DAO	1.0	reference	
	Only dynapenia	1.18	0.73–1.91	0.50
	Only abdominal obesity	1.30	1.09–1.56	0.004
	DAO	1.31	1.01–1.73	0.04
Sex	Male	1		
	Female	1.57	1.33–1.85	<0.0001
Age	One year increase	1.004	0.995–1.013	0.39
Ethnicity	Non-white	1		
	Whites	1.50	1.24–1.83	<0.0001
Education	Less than college	1		
	College	1.24	1.04–1.46	0.01
Yearly income	<50,000$	1		
	≥50,000$	1.01	0.85–1.20	0.88
Alcohol drinking in a typical week	One drink increase	1.07	1.01–1.13	0.01
Center for Epidemiologic Studies Depression Scale (CES-D)	One point increase	1.02	1.007–1.03	0.002
Physical activity scale for the Elderly (PASE)	One point increase	1.001	1.000–1.002	0.02
Number of medications	One point increase	1.04	1.006–1.07	0.02
Previous falls	No	1		
	Yes	3.26	2.72–3.90	<0.0001
Presence of radiological knee OA	No	1		
	Yes	1.04	0.89–1.21	0.65

OR, odds ratio; CI, confidence interval; OA, osteoarthritis. Model is mutually adjusted for all variables in the table. Incident falls (outcome) were those which were assessed during the follow-up period and referred to those that occurred since the baseline evaluation.

## 4. Discussion

In this large study of North American people affected by knee OA or at high risk for this condition with over 8 years of follow-up, we found that abdominal obesity and DAO, in particular, were associated with a higher incidence of falls, even after controlling for multiple confounders. On the contrary, dynapenia alone was not associated with incident falls.

Our analyses showed that being female, white, highly educated, drinking high levels of alcohol, depressed, and using an elevated number of medications were significant factors for falls, overall confirming previous research (29). The finding that DAO is associated with particularly high odds for falls is in line with the two previous longitudinal studies on this topic (13, 15). There are several plausible pathways that may explain why DAO is associated with greater risk of falling among middle-aged and older adults. First, the impaired muscular system in dynapenic abdominal obesity may lead to difficulties responding to postural correction with sufficient strength and speed, subsequently resulting in falls (13). Importantly, muscles in older individuals likely have limited ability to react to changing balance threats when compared to young adults, and unstable older adults present this to a greater extent than younger adults (30). Next, dynapenic abdominal obesity may increase the risk of falls owing to decreased postural stability caused by abdominal fat, as previously discussed. Those with abdominal obesity exhibit a greater proportion of body mass further away from the ankle axis of rotation and thus a larger ankle torque is required to counter the greater gravitational torque (31). This latter finding is also confirmed by our study that shows that abdominal obesity is a significant risk factor for falls, more than the presence of dynapenia alone. It is thus likely that both muscle strength and central obesity interact in the maintenance of postural control and thus the presence of dynapenic abdominal obesity increases risk of falls.

Findings from the present study and that of previous research (13) suggest that the implementation of interventions to prevent or reverse DAO may subsequently reduce fall risk. In this sense, it may be prudent to implement interventions to improve or maintain muscle strength and reduce excess central adiposity. Such interventions could focus on promotion of physical activity, strength training and proper nutrition (32). Finally, some interventions could be appropriate for both knee OA and decreasing the risk of falls including mind–body exercise, such as Tai-Chi (33).

The findings of our work must be interpreted within its limitations. First, several variables used in the analysis including falls were self-reported, potentially introducing a recall bias. Second, the OAI includes only people with knee OA or at high risk for this condition: therefore, this population is not representative of the general population. Third, handgrip strength was not available in the OAI dataset and, therefore, it is possible that people with knee OA may do badly on chair stands time due to pain. Fourth, the changes of anthropometric parameters and knee OA status were not included in the analyses, but they may affect our results. Finally, it is possible for DAO status or other control variables to have changed during the follow-up, but we did not assess these changes that could modify our results.

In conclusion, in the present study including a large representative sample of adults participating in the Osteoarthritis Initiative, over 8 years of follow-up, it was observed that DAO significantly increased risk of falls as well as the presence of abdominal obesity. Therefore, interventions to prevent or reverse dynapenic abdominal obesity may be beneficial for fall reduction, a phenomenon that afflict several people with important consequences.

## Data availability statement

The original contributions presented in the study are included in the article/supplementary materials, further inquiries can be directed to the corresponding author.

## Ethics statement

The OAI study was given full ethics approval by the institutional review board of the OAI Coordinating Center, at the University of California in San Francisco, CA, USA. The patients/participants provided their written informed consent to participate in this study.

## Author contributions

NV drafted the first version of the manuscript and made the statistical analysis. AK supervised the statistical analysis and critically revised the manuscript. PS drafted the first version of the manuscript. FB contributed to the data collection and drafted the first version of the manuscript. LD, MB, SS, NA-D, and LS critically revised the manuscript. All authors contributed to the article and approved the submitted version.

## Funding

The OAI is a public-private partnership comprised of five contracts (N01-AR-2-2258; N01-AR-2-2259; N01-AR-2-2260; N01-AR-2-2261; and N01-AR-2-2262) funded by the National Institutes of Health, a branch of the Department of Health and Human Services, and conducted by the OAI Study Investigators. Private funding partners include Merck Research Laboratories; Novartis Pharmaceuticals Corporation, GlaxoSmithKline; and Pfizer, Inc. The funder was not involved in the study design, collection, analysis, interpretation of data, the writing of this article, or the decision to submit it for publication. Private sector funding for the OAI is managed by the Foundation for the National Institutes of Health. This manuscript was prepared using an OAI public use data set and does not necessarily reflect the opinions or views of the OAI investigators, the NIH, or the private funding partners. The authors extend their appreciation to the Deputyship for Research & Innovation, Ministry of Education in Saudi Arabia for funding this research (IFKSURC-1-1602).

## Conflict of interest

The authors declare that the research was conducted in the absence of any commercial or financial relationships that could be construed as a potential conflict of interest.

## Publisher’s note

All claims expressed in this article are solely those of the authors and do not necessarily represent those of their affiliated organizations, or those of the publisher, the editors and the reviewers. Any product that may be evaluated in this article, or claim that may be made by its manufacturer, is not guaranteed or endorsed by the publisher.
